# Evaluation of different techniques for CT radiation profile width measurement

**DOI:** 10.1120/jacmp.v14i4.4122

**Published:** 2013-07-08

**Authors:** Steven R. Jackson, Salahuddin Ahmad, Yida Hu, Chun Ruan

**Affiliations:** ^1^ Department of Radiological Sciences University of Oklahoma Health Sciences Center Oklahoma City OK; ^2^ Department of Radiation Oncology University of Oklahoma Health Sciences Center Oklahoma City OK; ^3^ Department of Radiation Oncology University of Mississippi Medical Center Jackson MS USA

**Keywords:** computed radiography (CR), radiation profile, computed tomography (CT), radiochromic film, FWHM

## Abstract

This work has been conducted to demonstrate a procedure for using a Konica Minolta computed radiography (CR) system for the measurement of computed tomography (CT) radiation profile width, and to compare this method with conventional and GAFCHROMIC XR‐QA2 film measurements. The exposure and processing conditions of a Konica Minolta CR reader system were characterized to establish the relationship between exposure at the imaging plate (IP) and pixel value. A 6 cc ionization chamber was exposed at the isocenter of a CT scanner using 80 kVp, 0.4 sec with various mA settings. CR images were processed in fixed modes with various combinations of S and G values, establishing exposure and pixel value relationships. Appropriate exposure techniques and processing parameters were selected to avoid the saturation of the IP. Using the selected exposure and processing parameters, radiation profiles of various nominal collimation settings (40, 20, 10, and 5 mm) were acquired for measurement. Radiochromic film was characterized and utilized to compare with CR profiles and profiles obtained via conventional film. Appropriate exposures for both CR (80 kVp, large body filter, 4 and 8 mAs) and radiochromic films (120 kVp, large body filter, 300 mAs) were determined. Recommended CR processing settings (fixed mode with S=5 and G=1.81) were also determined. Compared to the conventional film results, the full width at half maximum (FWHM) results for CR agreed well within ±10%, while radiochromic film results showed maximum deviations of about 5%. In conclusion, FWHM of CT radiation profiles can be conveniently and accurately measured using a Konica Minolta CR system or XR‐QA2 film when appropriate exposure technique and processing parameters are used.

PACS numbers: 87.57.Q‐, 87.57.qp, 87.59.bd, 87.57.uq

## I. INTRODUCTION

In recent years, increased emphasis has been placed on the importance of quality assurance and patient radiation dose assessment in diagnostic radiology. Specifically, patient exposure to ionizing radiation as a result of computed tomography (CT) examinations has been of paramount concern. In the meantime, the general trend of radiology departments has been away from the use of conventional radiographic film. Prior to this trend, many quality assurance techniques were heavily reliant upon the use of film. The combination of increased pressure for more rigorous quality testing routines and decreased availability of film processors utilized in those testing routines has advanced the need for novel applications of technology in this arena. Over the past two decades, emerging technologies such as computed radiography (CR) have been available for clinical applications in contemporary radiology departments. As clinical imaging transitions to these new technologies, so too must quality assurance techniques. This necessitates studies to develop novel quality assurance applications and to evaluate the accuracy of these available technologies with respect to proven approaches.

Of particular interest to this research is the evaluation of CT radiation profile width. Accurately identifying the location of the primary radiation beam during CT scans is of obvious importance in patient protection and dose monitoring. Previously the measurement of CT radiation profile width relied heavily upon film.

CR shows initial promise for application in CT radiation profile measurement due simply to its widespread availability and associated familiarity. Previous works have shown the feasibility to measure CT radiation profile width using the CR system; however, these works have focused largely on Fuji CR systems.^1^, ^2^ The vendor‐specific image plate (IP) exposure index and the assignation of pixel value make implementation on non‐Fuji systems difficult, directly affecting the measurement of profile width. This work serves to address the lack of Konica Minolta CR system‐related characterization information in the current literature, as well as to define a general method for profile measurement using CR regardless of any specific CR vendor's formulaic details.

In order to verify these results, we have chosen to compare two commonly used standards to demonstrate the validity of this method for CT radiation profile width assessment: the established standard of conventional film and a recently popularized approach using GAFCHROMIC film. GAFCHROMIC film's ability to be cut to various sizes combined with its instantaneous color‐change result adds to this methodology's appeal and lends to the films usefulness in CT profile width measurements. However, as new films are introduced to the consumer market frequently, special care must be taken in calibration and evaluation of film response for specific film type prior to clinical use. Known factors that affect GAFCHROMIC film response in therapeutic energy ranges have been well‐published,^3^, ^4^ but few studies have reported on film response at diagnostic CT energy range. Additionally, despite the film's popularity for CT profile width measurement, no report assessing methods for determination of appropriate technique factors has been found. Therefore, this work also serves to establish an exposure and analysis methodology for GAFCHROMIC film's application in CT radiation profile measurement. The widespread usage of this type of film for CT radiation profile width measurement necessitates a study to compare its efficacy and accuracy against other techniques.

## II. MATERIALS AND METHODS

In this study, CT radiation profiles were obtained using a GE LightSpeed VCT scanner (GE Healthcare, Milwaukee, WI). Nominal beam width of 40(5×8i),20(5×4i),10(5×2i), and 5 (5×1i) mm were evaluated. In order to accurately determine relationships between the amount of incident radiation and the film or IP response, exposure levels for all relevant technique settings were measured at the beam isocenter using a 40 mm beam width and a 6 cc ionization chamber (Radcal 9010; Radcal Corporation, Monrovia, CA). Care was taken to ensure the 40 mm beam width provided irradiation of the full sensitive volume of the 6 cc ionization chamber.

### A. CT radiation profile width measurement using CR system

#### A.1 Exposure technique and processing parameter determination

This study utilized a Konica Minolta Regius 190 CR system (Konica Minolta Holdings, Inc., Tokyo, Japan). The chosen IP had dimensions of 14×17 inches and a resolution capability of 87.5μm.^(5)^ For Konica Minolta CR systems, three factors determine the final observed digital pixel value: the exposure the IP receives, a density correction parameter S, and a contrast value parameter G.

Defining the proper exposure range and scan field of view (SFOV) was a critical first step in establishing a protocol for radiation profile width measurement. Due to the fact that CT generally provides a much higher photon flux to the IP, technique factors must be chosen to avoid IP saturation. In light of this, the study began with a comprehensive analysis of CT scan parameters which produced the lowest possible exposure at isocenter with no IP saturation. Exposure measurements were taken starting at 80 kVp, 10 mA, and 0.4 sec rotation time, which is the lowest possible technique combination for the GE LightSpeed VCT scanner. Exposure readings were taken using a 40 mm nominal beam width from the 4 mAs starting point to 32 mAs at 2 mAs intervals for each of seven SFOVs available for this scanner. The examined SFOVs included small head, small body, pediatric head, pediatric body, medium head, medium body, and large body. Although the SFOV setting is typically used to reduce the effective radius of the reconstructed image, this setting also alters the added filtration,^(6)^ thus affecting the exposure delivered during the scanning procedure.

Once the exposure technique settings were determined for this study, we moved to defining appropriate processing parameters: S and G Analogous to conventional film latitude and speed, parameters S and G define a lookup table (LUT) which ultimately determines the relationship between pixel value and IP exposure. Processing IPs under a typical clinical processing mode automatically allows for S and G to be calculated for a given clinical application.^(7)^ In order to limit image postprocessing and avoid S and G value calculation, “fixed” mode processing was employed in this study. Fixed mode processing — which is available on most CR vendor platforms — allows the user to define processing parameters explicitly, avoiding the calculation step during processing and giving the user more control over the pixel value representation in the output image. S and G values to be used for profile measurements were determined experimentally in the following manner. Combinations of S and G were used to process plates minimally exposed to the previously discussed technique settings. The 4 to 32 mAs exposure range was used in conjunction with 14 different combinations of S and G in order to locate the saturation point for each set of values and determine most useful S, G, and mAs settings to be utilized in profile width testing. Location of two exposure points below the saturation point is essential in obtaining accurate results when using a two‐exposure technique,^(2)^ as exposing above saturation leads to artificial expansion of the profile width.^(1)^

#### A.2 Profile width measurement

**Figure 1:**
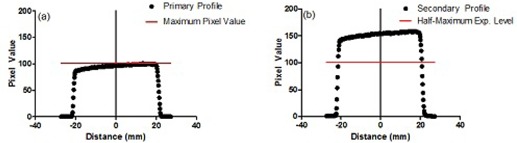
Example of profile measurement technique used. The measurement method requires two profiles to be obtained. The maximum pixel value in the first profile (a) becomes the half‐maximum pixel value in the second profile (b), which is exposed with twice the tube current.

Once appropriate exposure and processing parameters were established for the CR system, CT radiation profile measurements were performed. Profile exposures were made at the beam isocenter. After IPs were processed using the experimentally defined parameters, the final image was retrieved using General Electric Centricity picture archiving and communication system (GE Healthcare, Milwaukee, WI) and analyzed using ImageJ software (National Institutes of Health, Bethesda, MD). The ImageJ software allowed for profiles to be generated and full width at half maximum (FWHM) measurements to be accomplished. As outlined in previous works, the technique involves making two exposures of each nominal profile. The primary exposure should be at an exposure level that is one‐half of that for the secondary exposure.^(2)^ Once profiles are obtained, the maximum pixel value obtained from the primary profile indicates the half‐maximum exposure level in the secondary profile. Figure 1 gives an example of how FWHM has been determined. Figure 1(a) shows a primary 40 mm profile exposed at half the secondary exposure seen in Fig. 1(b). The red line in Fig. 1(a) shows the maximum pixel value in the primary profile. In Fig. 1(b), the same pixel value is indicated by the red line and now corresponds to the pixel value that represents the half‐maximum exposure level in the secondary profile. Thus, the FWHM can be determined at this location in the secondary profile.

### B. CT radiation profile width measurement using GAFCHROMIC film

#### B.1 GAFCHROMIC film calibration

This study utilized XR‐QA2 GAFCHROMIC film (International Specialty Products, Wayne, NJ; Lot #A10071002A) as another technique for radiation profile measurement. Similar to the CR method, a calibration study was performed first on film response to different exposure levels and beam energies, as well as to determine the useful technique factors for profile width measurement. Films were cut into 2.54cm×7.62cm strips, and 24 film strips were exposed at eight different exposure levels (controlled by varying tube current) at each of three energies: 80, 100, and 120 kVp. Each exposure was made at the beam isocenter using a nominal collimation of 40 mm and rotation time of 1 sec. Each exposure was repeated twice, once on the film strip and again using an ionization chamber at the same location to verify the exposure level for subsequent response curve determination. Films were then taken to an Epson 10000XL flatbed RGB scanner (Epson America Incorporated, Long Beach, CA) and scanned with a resolution of 300 divisions per inch (∼85μm) on reflective mode at 1 hour postirradiation. Films were placed on the scanner bed in precisely the same location to avoid any scanner inhomogeneity similar to what has been reported in previous works.^(8)^ Scanned images were analyzed using ImageJ software for mean red channel pixel response in a 1.8 cm^2^ region of interest (ROI) in the center of the exposed profile. This ROI size was chosen to complement the 40 mm collimation by being large enough to capture the usable data while remaining small enough to avoid the peripheral penumbra. In order to limit the effect of film nonuniformity, the ROI size remained consistent for all GAFCHROMIC film response measurements.^(9)^ The red channel image was used since it became evident that XR‐QA2 GAFCHROMIC film showed results typical of other radiochromic films in that maximum absorption is seen at red wavelength.^10^, ^11^, ^12^ After mean red channel pixel value (RCPV) was determined, a calibration curve was constructed. The established calibration curves were used to select technique factors for profile measurement. While many technique settings exist that are equally valid for use with profile measurement, avoidance of exposures that show evidence of film saturation is essential in maintaining accuracy of FWHM measurements.

#### B.2 Profile width measurement

**Figure 2:**
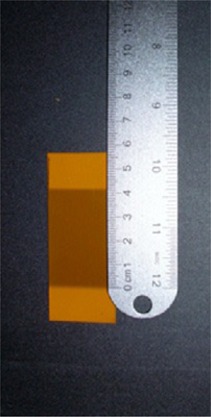
A 40 mm profile is measured visually using a metric ruler for verification purpose.

Films were again cut into 2.54cm×7.62cm strips and exposed at the same four nominal collimation widths as the CR profiles using the appropriate technique factors determined from the calibration measurements. Films were scanned at one hour postirradiation, and the red channel image was analyzed using ImageJ software, where maximum RCPV was obtained within a 1.8 cm^2^ ROI. Temperature and ambient light conditions were monitored and controlled during both exposure and readout phases to ensure no outside agents influenced the profile width measurement result. Profiles were obtained using ImageJ, and the pixel value that was half of the determined maximum RCPV served as the profile measurement location. Films were also measured visually with a metric ruler immediately postexposure for verification purposes, as shown in Fig. 2.

### C. CT radiation profile width measurement using conventional film

Conventional radiographic film measurements served as the standard to verify the accuracy of CR and GAFCHROMIC film results. Kodak X‐Omat V film (Eastman Kodak Company, Rochester, NY) was exposed at the beam isocenter at 120 kVp, 240 mAs using the same four nominal beam width settings as were used in CR and GAFCHROMIC film studies. The film was subsequently digitized using the same Epson 10000XL scanner used for GAFCHROMIC film analysis. Film digitization again allowed for construction and FWHM measurement of the corresponding profiles using ImageJ software.

## III. RESULTS

### A. CR Exposure technique and processing parameter determination

To avoid saturation of the CR image plates, which are not specifically designed for use with high exposures produced in CT, a study was carried out to determine technique factors which yield the lowest tube output at isocenter. The lowest exposures were seen using the “Large Body” SFOV, which produced exposures ranging from 14.08 mR (4 mAs) to 99.54 mR (32 mAs) for the 40 mm nominal beam width, 80 kVp, and rotation time of 0.4 sec.

**Figure 3:**
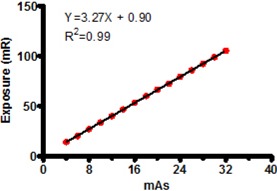
CT output linearity over the exposure range used for profile measurement with CR. Linearity is required for validity of the measurement technique.

The validity of the CR measurement technique relies on CT output linearity. Due to this requirement and the fact that the low technique factors used in the CR measurement are rarely used clinically, the output linearity was verified as shown in Fig. 3 ($R^{2}=0.99$).

**Figure 4:**
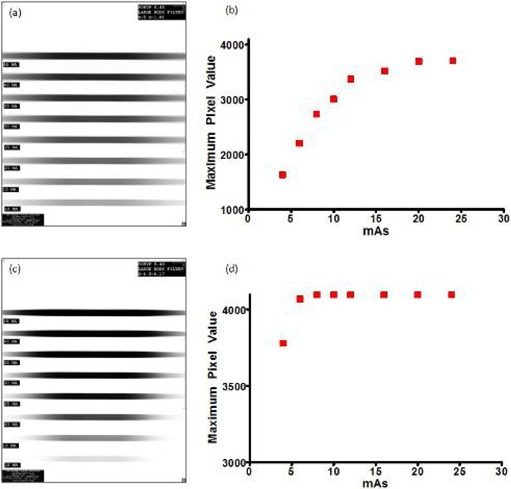
Corresponding visual and graphical difference in pixel value response of identically exposed IPs using S=5,G=1.81 ((a) and (b)), and S=1.39,G=0.60 ((c) and (d)).

**Figure 5:**
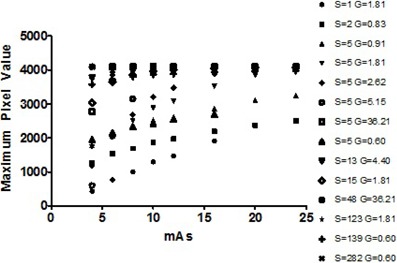
Maximum pixel value vs. mAs for all tested combinations of S and G.

Following the determination of exposure settings and verification of output linearity, a study of the processing response of the 14 S and G combinations (as described in the Methods section above) led to the determination of the exposure and processing settings to be used for profile measurement. Figure 4 shows eight 10 mm nominal profiles collected at 2 mAs increments between 4 and 18 mAs and processed using different sets of S and G; this exemplifies the procedure used to determine the appropriate exposure and processing settings for all profile measurements. Figure 4 presents the effect of different processing parameters on identically exposed IPs: ((a) S=5,G=1.81; (c) S=5,G=5.15). Figure 4 (b) and (d) give the corresponding maximum pixel value response. Note the presence of the saturation point in Fig. 4(b) allows for two mAs settings to be selected below the saturation point in order to accommodate the two‐exposure technique being used. However, the processing parameters used in Fig. 4(d) exhibit immediate saturation above the 4 mAs data point, which does not allow the proposed measurement technique to be used. Similar plots of pixel value versus mAs were constructed for all S and G combinations and can be seen compiled in Fig. 5. Following careful examination of the pixel value versus mAs relationships for all 14 processing combinations, appropriate parameters have been determined to be S=5 and G=1.81 in the fixed mode.

### B. GAFCHROMIC film calibration and exposure technique

**Figure 6:**
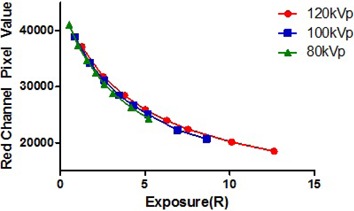
Pixel value to exposure calibration curve for GAFCHROMIC XR‐QA2 film at three tube voltages: 80, 100, and 120 kVp.

A calibration curve was constructed for the three energies of interest (Fig. 6). Avoidance of technique settings which show evidence of film saturation was of primary concern. Based on previously published results^(13)^ identifying kilovolt‐range energy dependence, it was also deemed prudent to keep energy consistent for radiochromic film measurements. A characterization of film response determined 120 kVp with 300 mAs to fit the criteria for an appropriate exposure setting for the profile width measurements using the XR‐QA2 GAFCHROMIC film. This technique produced a measured exposure of 7.535 R at the film location.

### C. Profile width measurements

Results for CR, GAFCHROMIC film, and conventional film profiles width are presented in Table 1. CR and GAFCHROMIC film results were compared to conventional film results for accuracy verification. CR measured profiles agree with conventional film within a maximum observed deviation of −9.7%. In slightly better agreement were the GAFCHROMIC film results, which show a maximum observed deviation from conventional film of −5.2%.

GAFCHROMIC film profile width was also measured directly with a metric ruler immediately after exposure. Table 2 shows the comparison between the FWHM measurements using ImageJ software after being scanned one hour after exposure and the simple visual measurement. The visual measurement maximally deviates from FWHM results by +1.2%.

**Table 1:** FWHM results of CT radiation profile width measurements for four nominal beam widths

*Nominal Beam Width (mm)*	*FWHM Conventional (mm)*	*FWHM GAFCHROMIC (mm)*	*FWHM CR (mm)*
40	43.3	43.2	42.3
20	23.5	22.2	21.7
10	13.0	13.0	12.4
5	8.0	8.0	7.2

**Table 2:** Results of GAFCHROMIC film obtained CT radiation profile width, measured as FWHM of computer‐generated profile and visually using a metric ruler

*Nominal Beam Width (mm)*	*FWHM GAFCHROMIC (mm)*	*Visual GAFCHROMIC (mm)*
40	43.2	43.5
20	22.2	22.5
10	13.0	13.0
5	8.0	8.0

## IV. DISCUSSION

CT radiation profile width measurement is of paramount importance to patient safety and radiation dose management. This work intends to establish a methodology for initial implementation of CR or GAFCHROMIC film‐based profile measurement routines in clinical setting. In addition, few studies have been published regarding characterization of Konica Minolta CR systems — making a reference for Konica CR users a necessity.

CR in general has largely taken the place of conventional film as the “workhorse” of the contemporary radiology department. Therefore, CR systems are both largely available and close in proximity to areas where CT testing is likely performed. CR results seen in Table 1 using a Konica Minolta system agree well with previously published results using a Fuji CR system.^(1)^ Keeping the plate minimally exposed, in addition to controlling processing parameters at known values, is the only way to avoid saturation and obtain reliable results. These precautions add necessary time and effort to the initial testing process and should be evaluated on a vendor‐by‐vendor and site‐by‐site basis. However, following the general procedure presented in this study, appropriate parameters can be established on a given system, allowing for subsequent measurements to be obtained more quickly, without the need to redetermine these values. Additionally, using CR for CT radiation profile measurements incurs no additional cost in departments with already established CR systems. These facts make CR an attractive choice for routine clinical use in the measurement of radiation profile width.

The GAFCHROMIC film results obtained through this work also show promise for the presented technique. Although GAFCHROMIC film use for radiation profile width measurement has already gained much popularity, this study provides technique recommendations, as well as affirmation of the accuracy of this technique in terms of comparison to established methods which has not been reported previously. Shown to agree well with conventional film in Table 1, this method is additionally attractive due to its ability to be made variable in size and its ease of use.

Resolution capability is an important factor to consider when evaluating different methods for CT profile measurement and directly affects the accuracy with which the profile measurement can be made. The maximum achievable resolution for CR will be dictated by plate construction. The resolution of film measurements is tied to digitizer capability. Since digitizers come in many different levels of quality, care should be taken to ensure digitizer capability and desired measurement accuracy can be matched before this methodology can be employed.

Table 2 results would indicate that, in the absence of an available scanner, an immediate visual measurement of the exposed film profile is an acceptable means for profile width measurement. Utilizing this method would certainly reduce measurement time and allow for CT radiation profile width measurements to be completed quite quickly. Table 2 results lend to the confidence of the visual measurement method which may be a more efficient measurement option in busy clinics. Although a ruler was used for visual measurements in this study, accuracy could be improved by making visual measurements using a magnifying loupe with a sub‐mm scale. Regardless of measurement device, an element of subjectivity may be introduced when making a visual measurement and results may vary slightly depending upon who is performing the test. Additionally, while film sheets can be cut as a conservational practice, films are not reusable as are CR IPs. Therefore, utilization of this technique introduces additional financial cost.

Results from this study showed good agreement for both CR and GAFCHROMIC film in comparison to conventional film. When implemented correctly, with a consistent preset protocol for exposure and analysis, both CR and GAFCHROMIC films have great potential to serve as measurement media for routine quality assurance testing of CT radiation profile width. Each method possesses both compelling properties and drawbacks to clinical implementation in the absence of conventional film.

The CR measurement technique employed in this study relies on CT output linearity at low exposure levels. Due to differences in technique settings, some of the lower exposures described in this study's CR profile measurements may not be achievable across all CT manufacturers’ platforms. For these cases, external filtration could be added or a similar methodology introduced in this work can be applied to determine the most appropriate technique factors. Specifically, a comprehensive review of output that begins with the lowest technique combination and includes multiple filtration settings is recommended for finding useful technique and processing parameter combinations for radiation profile measurement. In this way, the presented methodology can be used as guidance for measurements of radiation profile width using any combination of CT and CR manufacturers. Additionally, the presented measurement method can be employed in the absence of manufacturer‐provided equations of pixel value versus exposure relationships, which may not be easily attainable at all clinical sites.

## V. CONCLUSIONS

This work has established a methodology for initial implementation of CR or GAFCHROMIC film based profile measurement routines in clinical settings. Results showed the potential of CR systems, including Konica Minolta, to serve as accurate, viable means of replacement for conventional film in monitoring CT radiation profile width. Additionally, this work has highlighted the necessity of a comprehensive understanding of aspects relating to the exposure and processing of IPs when using CR as a measurement tool. Particularly, keeping IP exposure reasonable and choosing processing parameters which increase the dynamic range of the IP at that exposure level is essential. GAFCHROMIC films have been shown to be highly accurate means of radiation profile measurement, but may be cost‐prohibitive, especially in radiology departments with well‐established CR programs.

## ACKNOWLEDGMENTS

The authors would like to thank Dr. Hong Liu and Dr. Molly Wong for their assistance in IP processing and in the acquisition of useful references.
